# Telescopic Overdenture: A Case Report

**Published:** 2017-03

**Authors:** C. S. Shruthi, R. Poojya, Swati Ram, Anupama

**Affiliations:** 1 Professor, Department of Prosthodontics, M R Ambedkar Dental College and Hospital, Bangalore, Karnataka, India;; 2 Reader, Department of Prosthodontics, M R Ambedkar Dental College and Hospital, Bangalore, Karnataka, India;; 3 Post Graduate Student, Department of Prosthodontics, M R Ambedkar Dental College and Hospital, Bangalore, Karnataka, India;; 4 Post Graduate Student, Department of Prosthodontics, M R Ambedkar Dental College and Hospital, Bangalore, Karnataka, India

**Keywords:** overdenture, telescopic denture, double crown, crown and sleeve coping, telescopic overlay denture

## Abstract

**Patient:**

This report describes the case of a 68 year old female patient who presented with the chief complaint of difficulty in chewing and poor aesthetics due to missing teeth. The patient was interested in saving the remaining natural teeth and desired minimal tissue coverage from the prosthesis. After consideration of all the factors involved, it was deemed advisable to resort to a palate free maxillary telescopic complete denture and a mandibular removable partial denture.

**Discussion:**

Considering the age of the patient and the cost involved, implant supported prosthesis was ruled out as a treatment option for the patient. A telescopic denture was chosen as a favourable treatment option since it overcomes many of the problems posed by conventional complete dentures like progressive bone loss, lower stability and retention, loss of periodontal proprioception and low masticatory efficiency. It also provides minimal tissue coverage and better distribution of forces. Evaluation of occlusion, esthetics, phonetics and comfort after 24 hours, 1 week and 1 month of treatment showed that the patient was happy with the prosthesis and was able to speak and chew well.

**Conclusion:**

Telescopic overdentures have better retention and stability as compared to conventional complete dentures. They improve the chewing efficiency, patient comfort and also decrease the alveolar bone resorption. As such they are an excellent alternative to conventional complete denture treatment.

## INTRODUCTION

M. M. Devan stated, “It is perpetual preservation of what already exists and not the meticulous replacement of what is missing”.

When few teeth are remaining the options for replacement are tooth or tissue supported dentures, conventional fixed prosthesis or implant-supporting dentures. Dentures are often unsatisfactory for patients because of the lack of retention or excessive tissue coverage. Implants are often expensive and may require bone grafting for placement. In other cases, there may not be enough teeth present to support a fixed prosthesis. Furthermore, patients who have lost teeth due to poor oral hygiene may suffer the same problems with implants or bridges. In such cases, a removable prosthesis facilitates the maintenance of oral hygiene. A telescopic denture is an excellent alternative to overcome all of the above mentioned problems (1).

According to GPT, a telescopic denture is also called as an overdenture, which is defined as any removable dental prosthesis that covers and rests on one or more of the remaining natural teeth, on the roots of the natural teeth, and/or on the dental implants (2). It is a prosthesis which consists of a primary coping which is cemented to the abutments in a patient’s mouth and a secondary coping which is attached to the prosthesis which fits on the primary coping. Hence, it increases the retention and stability of the prosthesis. It is also called as overlay denture, overlay prosthesis and superimposed prosthesis (1, 3).

Telescopic crowns were initially introduced as retainers for the removable partial dentures at the beginning of the 20th century. Also known as a Double crown, a crown and sleeve coping or as Konuskrone (4), a German term that described a cone shaped design, these crowns are an effective means for retaining the RPDs and dentures. They transfer forces along the long axes of the abutment teeth, provide support and protection from the movements that dislodge the denture (3).

## OUTLINE OF THE CASE

A 68 year old female patient reported to the Department of Prosthodontics, Crown & Bridge and Implantology, M R Ambedkar Dental College and Hospital, Bengaluru, with the chief complaint of difficulty in chewing and poor aesthetics due to missing teeth.

On intraoral examination, teeth present were 14, 17, 24, 27, 34, 35, 37, 44 and 45. 35, 44 and 45 were grossly decayed showing grade II mobility. The other teeth were grossly decayed but firm. The edentulous span had favourable ridge with firmly attached keratinized mucosa. Radiographic examination of the remaining teeth revealed that 35, 44 and 45 had poor bone support while 14, 17, 24, 27, 34 and 37 had good bone support.

### Prosthetic Management

All the possible treatment options, including implant therapy, were given to the patient. The patient was interested in saving the remaining natural teeth and desired minimal tissue coverage from the prosthesis. After consideration of all the factors involved, it was deemed advisable to resort to a palate free maxillary telescopic complete denture and a mandibular removable partial denture.

**Figure 1:**
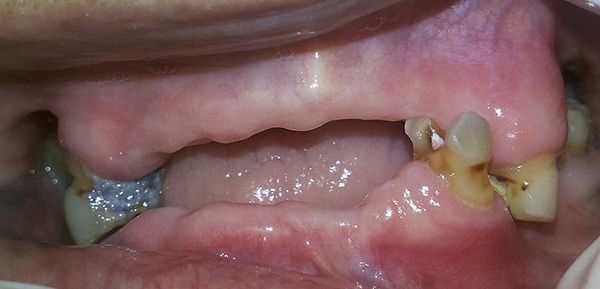
Pre-operative intraoral frontal view after extraction.

**Figure 2:**
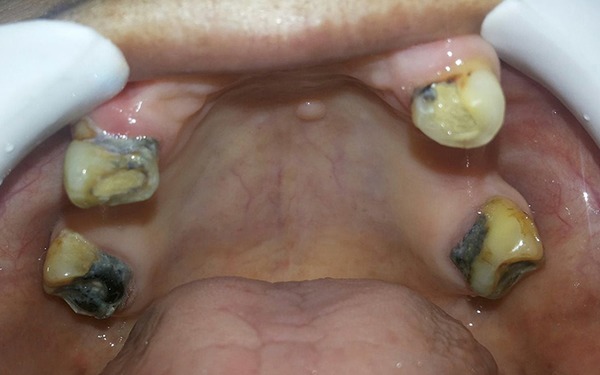
Pre-operative intraoral view of the maxillary arch after endodontic treatment.

**Figure 3:**
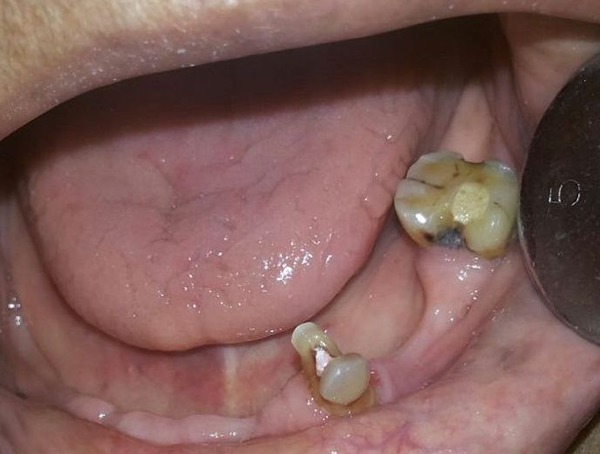
Pre-operative intraoral view of the mandibular arch after endodontic treatment.

Extraction of 35, 44 and 45 was done and the extraction sockets were allowed to heal (Figure 1). Primary impression of the maxillary and mandibular arches were made using irreversible hydrocolloid impression material and diagnostic casts were made with Type III gypsum product. Endodontic treatment of the remaining teeth was done, to use them as abutments for the maxillary telescopic complete denture and mandibular removable partial denture (Figure 2 and Figure 3). Temporary denture bases and wax occlusal rims were fabricated on the diagnostic casts. The occlusal rims were used to determine the vertical dimension of occlusion and occlusal plane, prior to tooth preparation.

**Figure 4:**
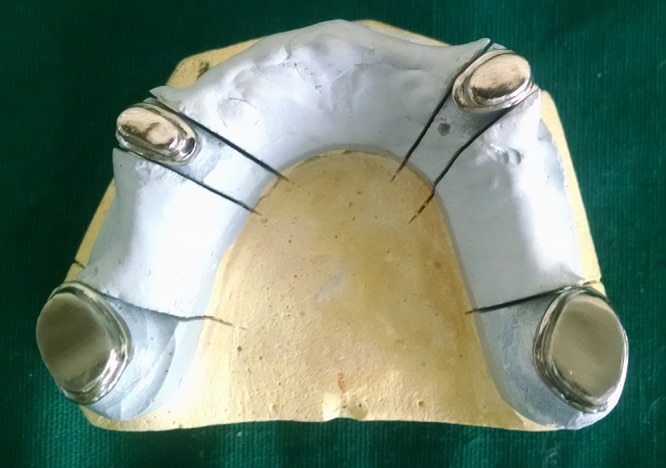
Primary metal copings.

**Figure 5:**
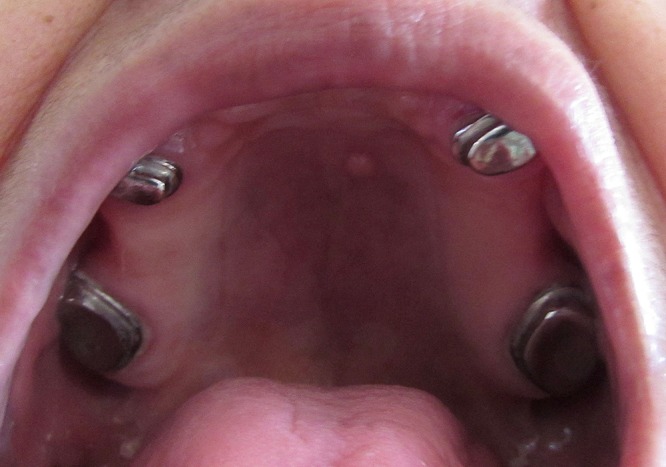
Intraoral view of primary copings cemented on 14, 17, 24 and 27.

**Figure 6:**
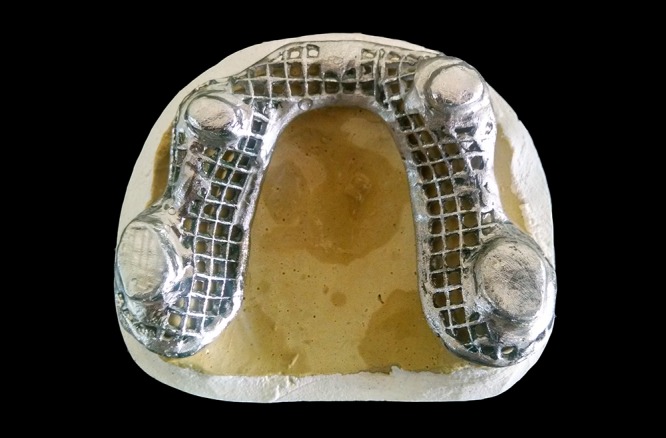
Metal framework with secondary copings ready for try-in.

In the maxillary arch, tooth preparation was done for 14, 17, 24 and 27 to receive primary copings. The occlusal rims acted as a guide for tooth preparation. After preparation of the abutments, impression was made using polyvinyl siloxane elastomeric impression material (putty and light body) by single step double mix technique. Primary copings were fabricated on the master cast obtained (Figure 4) and the fit of the copings were evaluated in the patient’s mouth, after which they were cemented on the abutments with glass ionomer cement (Figure 5). Construction of the maxillary metal framework was done on the master cast, following border molding and secondary impression. The metal framework included the secondary copings for the telescopic denture. The framework was tried in the patient’s mouth and necessary modifications were made (Figure 6).

In the mandibular arch, tooth preparation was done for 34 and 37 using the wax occlusal rims as a guide. Porcelain fused to metal crowns were fabricated cemented on the prepared teeth using glass ionomer cement. Border molding was done and secondary impression was made to obtain the master cast.

**Figure 7:**
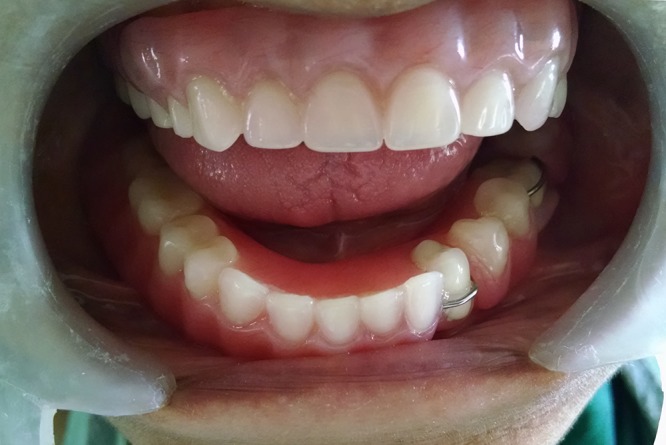
Intraoral view of the finished and polished maxillary telescopic overdenture and mandibular removable partial denture.

**Figure 8:**
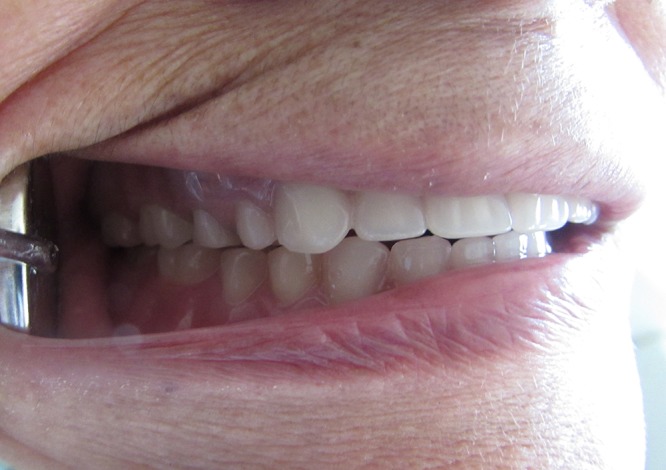
Right lateral view.

**Figure 9:**
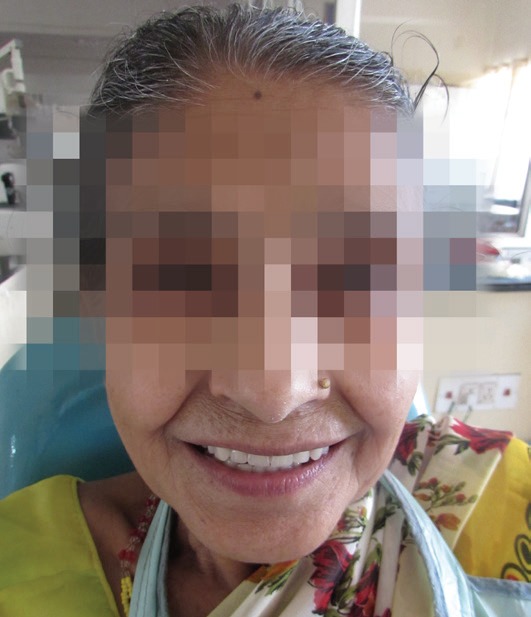
Post-placement view of the patient.

Maxillomandibular relation was recorded with wax occlusal rims on temporary denture bases fabricated on the mandibular master cast and the maxillary metal framework. Acrylic teeth were arranged and try-in was completed. After evaluating occlusion, phonetics and aesthetics, the final processing of the maxillary telescopic denture and mandibular removable partial denture was done. The prosthesis was polished and placed (Figure 7). Occlusion was assessed (Figure 8) and placement instructions were given. Post placement check up was done after 24 hours, 1 week and 1 month. The patient was happy with the prosthesis and was able to speak and chew well (Figure 9). The patient was instructed to attend recall visits every 6 months.

## DISCUSSION

Considering the age of the patient and the cost involved, implant supported prosthesis was ruled out as a treatment option for the patient. A telescopic denture was chosen as a favourable treatment option since it overcomes many of the problems posed by conventional complete dentures like progressive bone loss, lower stability and retention, loss of periodontal proprioception and low masticatory efficiency. It also provides minimal tissue coverage and better distribution of forces.

Owal *et al* found that 25% of RPDs fabricated were discarded during the first year due to unacceptable retention and stability of the prosthesis (5). This lack of retention and stability occurs because the residual alveolar ridge undergoes resorption in all directions following tooth loss (6). The resorption is stated to be rapid, progressive, irreversible and inevitable and has been well observed and documented in literature. It is equally well observed that bone is maintained around standing teeth and implants. Since overdenture therapy endeavours to preserve the few remaining natural teeth/tooth roots, it helps in the preservation of the alveolar ridge (7, 8). Robert J. Krum conducted a study to determine the amount of vertical residual bone loss in the anterior part of the maxillae and mandible in two groups of patients: One with complete maxillary dentures and mandibular overdentures and the other group with complete maxillary and mandibular conventional dentures. It was concluded that patients treated with complete maxillary dentures and mandibular overdentures demonstrated less vertical alveolar bone reduction than patients with complete maxillary and mandibular conventional dentures (9).

Longitudinal follow-up studies of 5-10 years report that conical crown-retained partial dentures have a lower failure rate compared to those retained with clasps or precision attachments (6). Teeth which are too weak to support a fixed partial denture and are unsuitable to support a removable partial denture can be conserved and suitably modified to act as abutments under overdentures for a good span of time (8). They provide tensile stimulation of the oblique periodontal fibres which leads to deposition of more bundle bone followed by decrease in abutment mobility. Telescopic prostheses are usually indicated only for patients with multiple abutments distributed bilaterally along the dental arch. The abutment teeth provide additional support to that supplied by the residual ridges. Stability is enhanced by the vertical component of the retained tooth/root. Proprioception through the periodontal fibres, gives the patient a sense of discrimination to touch and pressure, which is less with conventional complete dentures (1, 4, 6, 10).

Telescopic crowns consist of a primary telescopic coping which is permanently cemented to an abutment and a detachable secondary telescopic crown, rigidly connected to a detachable prosthesis. Copings protect the abutment from dental caries, thermal irritations and also provide retention and stabilization of the secondary crown. The secondary crown engages the primary coping to form a telescopic unit and serves as an anchor for the remainder of the prostheses. The tapered configuration of the contacting walls generates a compressive inter-surface tension based on wedging action. Tapering of the coping walls reduces retention between the unit elements. The smaller the degree of the taper, the greater the frictional retention of the retainer. The average wall taper commonly has a 6-degree angle. The copings are milled to exact configurations of taper angles of the walls with each other to create a common path of insertion for outer telescopic crowns of a retrievable superstructure (3, 4).

The primary advantage of a telescopic prosthesis is its retrievability. If the remaining dentition is in a state of transition, abutments splinted with FPDs can be a problem. A telescopic prosthesis is a more versatile alternative for these patients because the prosthesis can be repaired without reconstruction of the entire superstructure. Precautions to prevent damage to the denture during cleaning should be given to the patient since distortion of an outer telescopic crown can render reduce the retention of the prosthesis. Telescopic dentures also promote oral hygiene and periodontal health because the abutments are more accessible for oral hygiene (4, 11).

## CONCLUSION

Although there are increased costs and appointments associated with this technique, telescopic overdentures are a superior health service compared to the conventional complete denture. Tooth supported removable overdentures with telescopic crowns provide better retention, stability, support, stable occlusion and proprioception which increases chewing efficiency and phonetics. It also decreases the rate of residual ridge resorption due to conversion of compressive forces into tensile and better stress distribution. Even with the increased use of implants for overdenture therapy, tooth/root supported telescopic overdenture still remains an excellent treatment modality.
